# 6-(3,5-Dimethyl­benz­yl)-5-ethyl-1-[(2-phen­oxy­eth­oxy)meth­yl]-1,2,3,4-tetra­hydro­pyrimidine-2,4-dione

**DOI:** 10.1107/S1600536811039821

**Published:** 2011-10-05

**Authors:** Nasser R. El-Brollosy, Ali A. El-Emam, Omar A. Al-Deeb, Seik Weng Ng

**Affiliations:** a Department of Pharmaceutical Chemistry, College of Pharmacy, King Saud University, PO Box 2457 Riyadh 11451, Saudi Arabia; b Department of Chemistry, University of Malaya, 50603 Kuala Lumpur, Malaysia; c Chemistry Department, Faculty of Science, King Abdulaziz University, PO Box 80203 Jeddah, Saudi Arabia

## Abstract

The six-membered ring of the uracil part of the title compound, C_24_H_28_N_2_O_4_, is nearly planar (r.m.s. deviation = 0.013 Å); the aromatic ring of the 3,5-dimethyl­benzyl substitutent is aligned at 85.4 (1)° with respect to this mean plane. The phenyl ring of the substituent at the 1-position takes up two orientations in a 1:1 ratio. In the crystal, two mol­ecules are liked by a pair of N—H⋯O hydrogen bonds, generating a centrosymmetric hydrogen-bonded dimer.

## Related literature

For the background to our studies on anti­viral HIV chemicals, see: El-Brollosy *et al.* (2007 ▶, 2008 ▶, 2009 ▶).
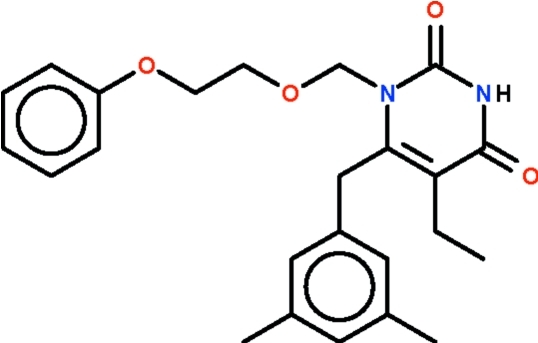


## Experimental

### 

#### Crystal data

C_24_H_28_N_2_O_4_
*M* *_r_* = 408.48
Monoclinic, 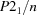

*a* = 16.1116 (6) Å
*b* = 4.8211 (2) Å
*c* = 27.5125 (10) Å
β = 92.574 (3)°
*V* = 2134.90 (14) Å^3^
*Z* = 4
Cu *K*α radiation
μ = 0.70 mm^−1^
*T* = 100 K
0.20 × 0.10 × 0.05 mm

#### Data collection

Agilent SuperNova Dual diffractometer with an Atlas detector
Absorption correction: multi-scan (*CrysAlis PRO*; Agilent, 2010 ▶) *T*_min_ = 0.873, *T*_max_ = 0.966
6587 measured reflections
4115 independent reflections
2940 reflections with *I* > 2σ(*I*)
*R*_int_ = 0.036

#### Refinement

*R*[*F* ^2^ > 2σ(*F* ^2^)] = 0.048
*wR*(*F* ^2^) = 0.130
*S* = 1.03
4115 reflections
272 parameters
1 restraint
H atoms treated by a mixture of independent and constrained refinement
Δρ_max_ = 0.20 e Å^−3^
Δρ_min_ = −0.33 e Å^−3^

### 

Data collection: *CrysAlis PRO* (Agilent, 2010 ▶); cell refinement: *CrysAlis PRO*; data reduction: *CrysAlis PRO*; program(s) used to solve structure: *SHELXS97* (Sheldrick, 2008 ▶); program(s) used to refine structure: *SHELXL97* (Sheldrick, 2008 ▶); molecular graphics: *X-SEED* (Barbour, 2001 ▶); software used to prepare material for publication: *publCIF* (Westrip, 2010 ▶).

## Supplementary Material

Crystal structure: contains datablock(s) global, I. DOI: 10.1107/S1600536811039821/xu5341sup1.cif

Structure factors: contains datablock(s) I. DOI: 10.1107/S1600536811039821/xu5341Isup2.hkl

Supplementary material file. DOI: 10.1107/S1600536811039821/xu5341Isup3.cml

Additional supplementary materials:  crystallographic information; 3D view; checkCIF report

## Figures and Tables

**Table 1:** Hydrogen-bond geometry (Å, °)

*D*—H⋯*A*	*D*—H	H⋯*A*	*D*⋯*A*	*D*—H⋯*A*
N1—H1⋯O2^i^	0.92 (3)	1.94 (3)	2.859 (2)	173 (2)

Symmetry code: (i) 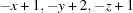.

**Fig. 1.**
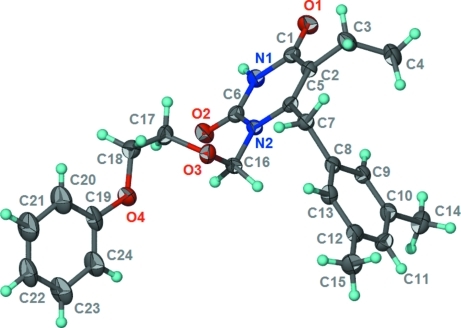
Thermal ellipsoid plot (Barbour, 2001) of C24H28N2O4 at the 70% probability level; hydrogen atoms are drawn as spheres of arbitrary radius. The disorder in the phenyl ring is not shown.

## supplementary crystallographic information

### Comment

Non-nucleoside reverse transcriptase inhibitors (NNRTIs) are chemicals for treating the human immunodeficiency virus (HIV), and the synthesis of such compounds represents a major thrust of our studies. We have synthesized several analogs of emivirine, TNK-651 and GCA-186 (El-Brollosy *et al.*, 2007; 2008; 2009). We have synthesized the title compound (Scheme I) as a GCA-186 analog but with phenoxyethoxymethyl and ethyl substituents instead of ethoxymethyl and isopropyl at the 1- and 5-positions. Its antiviral activity against HIV will be reported elsewhere.

The six-membered ring of the uracil part is planar. The aromatic ring of the 3,5-dimethylbenzyl substitutent is aligned at 85.4 (1)° with respect to this plane. The phenyl ring of the substituent at the 1-position is disordered over two orientations in a 1:1 ratio (Fig. 1). Two molecules are liked by an N–H···O hydrogen bond to generate a centrosymmetric hydrogen-bonded dimer (Table 1).

### Experimental

5-Ethyl-6-(3,5-dimethylbenzyl)uracil (0.258 g,1 mmol) was stirred in anhydrous acetontrile (15 ml) under nitrogen and *N*,*O*-bis(trimethylsilyl)acetamide (0.87 ml, 3.5 mol) was added. The clear solution -50° C and trimethylsilyl trifluoromethanesulfonate (0.18 ml, 1 mmol) was added followed by the dropwise addition of bis-(phenoxyethyloxy)methane (0.576 mg, 2 mmol). The mixture was stirred at room temperature for 4 h. The reaction was quenched with saturate sodium bicarbonate soluiton (5 ml). The solvent was evaporated under reduced pressure. The residue was extracted with ether (3 *x* 50 ml); the combined organic fractions were dried over magnesium sulfate. The solvent was removed and the residue was chromatographed on silica gel column with chloroform to afford a white solid. This was recrystallized from ethanol to yield the title compound as colorless crystals.

### Refinement

Carbon-bound H-atoms were placed in calculated positions [C—H 0.95 to 0.98 Å, *U*_iso_(H) 1.2 to 1.5*U*_eq_(C)] and were included in the refinement in the riding model approximation.

The amino H-atom was located in a difference Fourier map, and was freely refined.

The phenyl ring is disordered over two positions; the disorder could not be refined and was regarded as a is a 1:1 type of disorder. The ring was refined as a rigid hexagon of 1.39 Å sides. The temperature factors of the primed atoms were set to those of the unprimed ones; the pair of *O*–C_phenyl_ distances were restrained to within 0.01 of each other.

### Crystal data

**Table:**

C_24_H_28_N_2_O_4_	*F*(000) = 872
*M**_r_* = 408.48	*D*_x_ = 1.271 Mg m^−^^3^
Monoclinic, *P*2_1_/*n*	Cu *K*α radiation, λ = 1.54184 Å
Hall symbol: -P 2yn	Cell parameters from 1976 reflections
*a* = 16.1116 (6) Å	θ = 2.7–74.3°
*b* = 4.8211 (2) Å	µ = 0.70 mm^−^^1^
*c* = 27.5125 (10) Å	*T* = 100 K
β = 92.574 (3)°	Prism, colorless
*V* = 2134.90 (14) Å^3^	0.20 × 0.10 × 0.05 mm
*Z* = 4	

### Data collection

**Table:**

Agilent SuperNova Dual diffractometer with an Atlas detector	4115 independent reflections
Radiation source: SuperNova (Cu) X-ray Source	2940 reflections with *I* > 2σ(*I*)
Mirror	*R*_int_ = 0.036
Detector resolution: 10.4041 pixels mm^-1^	θ_max_ = 74.4°, θ_min_ = 3.1°
ω scans	*h* = −17→19
Absorption correction: multi-scan (*CrysAlis PRO*; Agilent, 2010)	*k* = −5→5
*T*_min_ = 0.873, *T*_max_ = 0.966	*l* = −19→34
6587 measured reflections	

### Refinement

**Table:**

Refinement on *F*^2^	Primary atom site location: structure-invariant direct methods
Least-squares matrix: full	Secondary atom site location: difference Fourier map
*R*[*F*^2^ > 2σ(*F*^2^)] = 0.048	Hydrogen site location: inferred from neighbouring sites
*wR*(*F*^2^) = 0.130	H atoms treated by a mixture of independent and constrained refinement
*S* = 1.03	*w* = 1/[σ^2^(*F*_o_^2^) + (0.0602*P*)^2^] where *P* = (*F*_o_^2^ + 2*F*_c_^2^)/3
4115 reflections	(Δ/σ)_max_ = 0.001
272 parameters	Δρ_max_ = 0.20 e Å^−^^3^
1 restraint	Δρ_min_ = −0.33 e Å^−^^3^

### Fractional atomic coordinates and isotropic or equivalent isotropic displacement parameters (Å^2^)

**Table:**

	*x*	*y*	*z*	*U*_iso_*/*U*_eq_	Occ. (<1)
N1	0.41200 (10)	0.7342 (4)	0.49973 (5)	0.0225 (4)	
H1	0.4438 (15)	0.827 (5)	0.4779 (8)	0.048 (7)*	
N2	0.38205 (9)	0.6427 (3)	0.58001 (5)	0.0188 (3)	
O1	0.34103 (8)	0.5312 (3)	0.43648 (4)	0.0288 (3)	
O2	0.48232 (8)	0.9577 (3)	0.56125 (4)	0.0268 (3)	
O3	0.44380 (7)	0.4797 (3)	0.65593 (4)	0.0248 (3)	
O4	0.56832 (8)	0.5868 (3)	0.72760 (5)	0.0324 (4)	
C1	0.35269 (11)	0.5509 (4)	0.48060 (6)	0.0207 (4)	
C2	0.30735 (11)	0.3942 (4)	0.51623 (6)	0.0200 (4)	
C3	0.24618 (11)	0.1866 (5)	0.49479 (7)	0.0265 (5)	
H3A	0.2372	0.0400	0.5192	0.032*	
H3B	0.2706	0.0976	0.4663	0.032*	
C4	0.16242 (13)	0.3118 (6)	0.47889 (8)	0.0397 (6)	
H4A	0.1283	0.1710	0.4618	0.060*	
H4B	0.1711	0.4688	0.4571	0.060*	
H4C	0.1341	0.3762	0.5076	0.060*	
C5	0.32268 (11)	0.4450 (4)	0.56414 (6)	0.0187 (4)	
C6	0.42908 (11)	0.7878 (4)	0.54791 (6)	0.0204 (4)	
C7	0.27874 (11)	0.2963 (4)	0.60376 (6)	0.0208 (4)	
H7A	0.3209	0.2359	0.6288	0.025*	
H7B	0.2525	0.1271	0.5896	0.025*	
C8	0.21249 (11)	0.4626 (4)	0.62863 (6)	0.0201 (4)	
C9	0.16152 (11)	0.6537 (4)	0.60390 (6)	0.0210 (4)	
H9	0.1699	0.6903	0.5705	0.025*	
C10	0.09878 (11)	0.7921 (4)	0.62691 (7)	0.0244 (4)	
C11	0.08768 (12)	0.7369 (4)	0.67617 (7)	0.0267 (5)	
H11	0.0455	0.8325	0.6925	0.032*	
C12	0.13705 (11)	0.5453 (4)	0.70170 (6)	0.0244 (4)	
C13	0.19980 (11)	0.4113 (4)	0.67758 (6)	0.0229 (4)	
H13	0.2346	0.2825	0.6949	0.028*	
C14	0.04326 (12)	0.9954 (5)	0.59964 (8)	0.0326 (5)	
H14A	0.0550	0.9909	0.5650	0.049*	
H14B	0.0535	1.1826	0.6125	0.049*	
H14C	−0.0150	0.9453	0.6037	0.049*	
C15	0.12262 (13)	0.4790 (5)	0.75430 (7)	0.0335 (5)	
H15A	0.1071	0.6487	0.7713	0.050*	
H15B	0.1736	0.4029	0.7698	0.050*	
H15C	0.0778	0.3424	0.7560	0.050*	
C16	0.39924 (11)	0.6934 (4)	0.63250 (6)	0.0212 (4)	
H16A	0.4311	0.8682	0.6365	0.025*	
H16B	0.3458	0.7180	0.6484	0.025*	
C17	0.53086 (11)	0.4743 (5)	0.64639 (7)	0.0297 (5)	
H17A	0.5493	0.6616	0.6368	0.036*	
H17B	0.5406	0.3453	0.6192	0.036*	
C18	0.57914 (12)	0.3821 (5)	0.69086 (7)	0.0317 (5)	
H18A	0.5587	0.1998	0.7018	0.038*	
H18B	0.6387	0.3638	0.6840	0.038*	
C19	0.6138 (7)	0.580 (2)	0.7701 (2)	0.0314 (7)	0.50
C20	0.6750 (6)	0.3880 (17)	0.7837 (2)	0.0428 (16)	0.50
H20	0.6865	0.2373	0.7628	0.051*	0.50
C21	0.7193 (4)	0.4169 (15)	0.8279 (2)	0.0473 (14)	0.50
H21	0.7611	0.2860	0.8373	0.057*	0.50
C22	0.7025 (4)	0.6374 (15)	0.85853 (18)	0.0386 (18)	0.50
H22	0.7328	0.6572	0.8888	0.046*	0.50
C23	0.6413 (6)	0.8290 (14)	0.8449 (2)	0.0400 (17)	0.50
H23	0.6298	0.9797	0.8658	0.048*	0.50
C24	0.5969 (7)	0.800 (2)	0.8007 (2)	0.0365 (14)	0.50
H24	0.5551	0.9309	0.7914	0.044*	0.50
C19'	0.6218 (7)	0.570 (2)	0.7688 (2)	0.0314 (7)	0.50
C20'	0.6910 (6)	0.3974 (18)	0.7719 (2)	0.0428 (16)	0.50
H20'	0.7033	0.2811	0.7453	0.051*	0.50
C21'	0.7423 (4)	0.3952 (17)	0.8139 (2)	0.0473 (14)	0.50
H21'	0.7896	0.2774	0.8160	0.057*	0.50
C22'	0.7244 (4)	0.5654 (18)	0.85281 (19)	0.0386 (18)	0.50
H22'	0.7594	0.5639	0.8815	0.046*	0.50
C23'	0.6552 (5)	0.7379 (15)	0.84974 (19)	0.0400 (17)	0.50
H23'	0.6429	0.8542	0.8764	0.048*	0.50
C24'	0.6039 (6)	0.740 (2)	0.8077 (2)	0.0365 (14)	0.50
H24'	0.5566	0.8579	0.8056	0.044*	0.50

### Atomic displacement parameters (Å^2^)

**Table:**

	*U*^11^	*U*^22^	*U*^33^	*U*^12^	*U*^13^	*U*^23^
N1	0.0214 (8)	0.0306 (10)	0.0160 (7)	−0.0053 (7)	0.0047 (6)	0.0009 (7)
N2	0.0188 (7)	0.0235 (9)	0.0142 (7)	−0.0030 (7)	0.0028 (6)	−0.0016 (7)
O1	0.0305 (7)	0.0412 (9)	0.0151 (6)	−0.0050 (7)	0.0035 (5)	−0.0015 (6)
O2	0.0257 (7)	0.0348 (8)	0.0202 (6)	−0.0111 (6)	0.0040 (5)	0.0003 (6)
O3	0.0187 (6)	0.0374 (8)	0.0182 (6)	0.0007 (6)	0.0013 (5)	0.0028 (6)
O4	0.0285 (7)	0.0444 (9)	0.0235 (7)	0.0064 (7)	−0.0068 (6)	−0.0081 (7)
C1	0.0197 (9)	0.0259 (11)	0.0166 (9)	0.0023 (8)	0.0032 (7)	0.0006 (8)
C2	0.0184 (9)	0.0216 (10)	0.0200 (9)	−0.0009 (7)	0.0019 (7)	−0.0011 (8)
C3	0.0287 (11)	0.0323 (12)	0.0186 (9)	−0.0090 (9)	0.0009 (8)	−0.0010 (9)
C4	0.0278 (11)	0.0587 (17)	0.0320 (11)	−0.0065 (11)	−0.0045 (9)	−0.0122 (12)
C5	0.0167 (8)	0.0205 (10)	0.0191 (8)	0.0016 (7)	0.0028 (7)	0.0000 (8)
C6	0.0178 (9)	0.0252 (10)	0.0183 (9)	0.0000 (8)	0.0035 (7)	0.0013 (8)
C7	0.0201 (9)	0.0234 (10)	0.0191 (9)	−0.0024 (8)	0.0031 (7)	−0.0005 (8)
C8	0.0176 (8)	0.0233 (10)	0.0196 (9)	−0.0055 (7)	0.0038 (7)	−0.0007 (8)
C9	0.0194 (9)	0.0253 (10)	0.0185 (9)	−0.0047 (8)	0.0021 (7)	0.0011 (8)
C10	0.0206 (9)	0.0243 (11)	0.0281 (10)	−0.0030 (8)	−0.0001 (8)	−0.0005 (9)
C11	0.0211 (9)	0.0309 (12)	0.0286 (10)	−0.0014 (8)	0.0069 (8)	−0.0041 (9)
C12	0.0213 (9)	0.0336 (12)	0.0186 (9)	−0.0064 (9)	0.0038 (7)	−0.0019 (9)
C13	0.0211 (9)	0.0286 (11)	0.0191 (9)	−0.0024 (8)	0.0010 (7)	0.0042 (8)
C14	0.0253 (10)	0.0321 (12)	0.0405 (12)	0.0011 (9)	0.0010 (9)	0.0029 (11)
C15	0.0292 (10)	0.0508 (15)	0.0210 (10)	−0.0065 (10)	0.0078 (8)	−0.0011 (10)
C16	0.0200 (9)	0.0281 (11)	0.0156 (8)	−0.0024 (8)	0.0022 (7)	−0.0029 (8)
C17	0.0177 (9)	0.0481 (14)	0.0235 (10)	−0.0014 (9)	0.0032 (7)	−0.0040 (10)
C18	0.0258 (10)	0.0389 (13)	0.0301 (11)	0.0067 (9)	−0.0033 (8)	−0.0086 (10)
C19	0.0248 (19)	0.0451 (15)	0.0237 (10)	−0.0112 (13)	−0.0055 (10)	0.0059 (10)
C20	0.040 (4)	0.0552 (19)	0.032 (3)	−0.001 (2)	−0.012 (3)	0.009 (2)
C21	0.039 (4)	0.063 (2)	0.038 (4)	0.002 (3)	−0.013 (2)	0.009 (3)
C22	0.030 (3)	0.058 (4)	0.0265 (18)	−0.013 (3)	−0.008 (2)	0.012 (2)
C23	0.042 (3)	0.059 (5)	0.0179 (14)	−0.010 (3)	−0.0079 (14)	0.011 (2)
C24	0.0328 (18)	0.062 (4)	0.0146 (19)	−0.0076 (19)	−0.0034 (13)	0.006 (2)
C19'	0.0248 (19)	0.0451 (15)	0.0237 (10)	−0.0112 (13)	−0.0055 (10)	0.0059 (10)
C20'	0.040 (4)	0.0552 (19)	0.032 (3)	−0.001 (2)	−0.012 (3)	0.009 (2)
C21'	0.039 (4)	0.063 (2)	0.038 (4)	0.002 (3)	−0.013 (2)	0.009 (3)
C22'	0.030 (3)	0.058 (4)	0.0265 (18)	−0.013 (3)	−0.008 (2)	0.012 (2)
C23'	0.042 (3)	0.059 (5)	0.0179 (14)	−0.010 (3)	−0.0079 (14)	0.011 (2)
C24'	0.0328 (18)	0.062 (4)	0.0146 (19)	−0.0076 (19)	−0.0034 (13)	0.006 (2)

### Geometric parameters (Å, °)

**Table:**

N1—C6	1.366 (2)	C13—H13	0.9500
N1—C1	1.388 (2)	C14—H14A	0.9800
N1—H1	0.92 (3)	C14—H14B	0.9800
N2—C6	1.380 (2)	C14—H14C	0.9800
N2—C5	1.406 (2)	C15—H15A	0.9800
N2—C16	1.479 (2)	C15—H15B	0.9800
O1—C1	1.224 (2)	C15—H15C	0.9800
O2—C6	1.230 (2)	C16—H16A	0.9900
O3—C16	1.396 (2)	C16—H16B	0.9900
O3—C17	1.439 (2)	C17—C18	1.488 (3)
O4—C19	1.352 (6)	C17—H17A	0.9900
O4—C19'	1.395 (6)	C17—H17B	0.9900
O4—C18	1.429 (2)	C18—H18A	0.9900
C1—C2	1.459 (2)	C18—H18B	0.9900
C2—C5	1.352 (2)	C19—C20	1.3900
C2—C3	1.506 (3)	C19—C24	1.3900
C3—C4	1.525 (3)	C20—C21	1.3900
C3—H3A	0.9900	C20—H20	0.9500
C3—H3B	0.9900	C21—C22	1.3900
C4—H4A	0.9800	C21—H21	0.9500
C4—H4B	0.9800	C22—C23	1.3900
C4—H4C	0.9800	C22—H22	0.9500
C5—C7	1.508 (2)	C23—C24	1.3900
C7—C8	1.522 (2)	C23—H23	0.9500
C7—H7A	0.9900	C24—H24	0.9500
C7—H7B	0.9900	C19'—C20'	1.3900
C8—C9	1.392 (3)	C19'—C24'	1.3900
C8—C13	1.393 (2)	C20'—C21'	1.3900
C9—C10	1.388 (3)	C20'—H20'	0.9500
C9—H9	0.9500	C21'—C22'	1.3900
C10—C11	1.401 (3)	C21'—H21'	0.9500
C10—C14	1.504 (3)	C22'—C23'	1.3900
C11—C12	1.389 (3)	C22'—H22'	0.9500
C11—H11	0.9500	C23'—C24'	1.3900
C12—C13	1.393 (3)	C23'—H23'	0.9500
C12—C15	1.510 (2)	C24'—H24'	0.9500
			
C6—N1—C1	126.42 (16)	H14B—C14—H14C	109.5
C6—N1—H1	116.5 (15)	C12—C15—H15A	109.5
C1—N1—H1	117.1 (15)	C12—C15—H15B	109.5
C6—N2—C5	122.04 (15)	H15A—C15—H15B	109.5
C6—N2—C16	117.20 (15)	C12—C15—H15C	109.5
C5—N2—C16	120.70 (15)	H15A—C15—H15C	109.5
C16—O3—C17	114.49 (15)	H15B—C15—H15C	109.5
C19—O4—C18	121.0 (4)	O3—C16—N2	113.50 (15)
C19'—O4—C18	116.3 (4)	O3—C16—H16A	108.9
O1—C1—N1	119.88 (17)	N2—C16—H16A	108.9
O1—C1—C2	124.54 (18)	O3—C16—H16B	108.9
N1—C1—C2	115.58 (15)	N2—C16—H16B	108.9
C5—C2—C1	119.08 (17)	H16A—C16—H16B	107.7
C5—C2—C3	126.12 (17)	O3—C17—C18	109.48 (15)
C1—C2—C3	114.80 (16)	O3—C17—H17A	109.8
C2—C3—C4	113.85 (18)	C18—C17—H17A	109.8
C2—C3—H3A	108.8	O3—C17—H17B	109.8
C4—C3—H3A	108.8	C18—C17—H17B	109.8
C2—C3—H3B	108.8	H17A—C17—H17B	108.2
C4—C3—H3B	108.8	O4—C18—C17	107.43 (17)
H3A—C3—H3B	107.7	O4—C18—H18A	110.2
C3—C4—H4A	109.5	C17—C18—H18A	110.2
C3—C4—H4B	109.5	O4—C18—H18B	110.2
H4A—C4—H4B	109.5	C17—C18—H18B	110.2
C3—C4—H4C	109.5	H18A—C18—H18B	108.5
H4A—C4—H4C	109.5	O4—C19—C20	126.9 (6)
H4B—C4—H4C	109.5	O4—C19—C24	113.0 (6)
C2—C5—N2	121.15 (16)	C20—C19—C24	120.0
C2—C5—C7	123.20 (17)	C19—C20—C21	120.0
N2—C5—C7	115.65 (15)	C19—C20—H20	120.0
O2—C6—N1	121.52 (16)	C21—C20—H20	120.0
O2—C6—N2	122.85 (16)	C22—C21—C20	120.0
N1—C6—N2	115.63 (16)	C22—C21—H21	120.0
C5—C7—C8	115.86 (16)	C20—C21—H21	120.0
C5—C7—H7A	108.3	C23—C22—C21	120.0
C8—C7—H7A	108.3	C23—C22—H22	120.0
C5—C7—H7B	108.3	C21—C22—H22	120.0
C8—C7—H7B	108.3	C24—C23—C22	120.0
H7A—C7—H7B	107.4	C24—C23—H23	120.0
C9—C8—C13	118.74 (17)	C22—C23—H23	120.0
C9—C8—C7	122.65 (16)	C23—C24—C19	120.0
C13—C8—C7	118.53 (17)	C23—C24—H24	120.0
C10—C9—C8	121.43 (17)	C19—C24—H24	120.0
C10—C9—H9	119.3	C20'—C19'—C24'	120.0
C8—C9—H9	119.3	C20'—C19'—O4	123.3 (6)
C9—C10—C11	118.53 (18)	C24'—C19'—O4	116.7 (6)
C9—C10—C14	120.98 (17)	C19'—C20'—C21'	120.0
C11—C10—C14	120.49 (18)	C19'—C20'—H20'	120.0
C12—C11—C10	121.36 (18)	C21'—C20'—H20'	120.0
C12—C11—H11	119.3	C20'—C21'—C22'	120.0
C10—C11—H11	119.3	C20'—C21'—H21'	120.0
C11—C12—C13	118.63 (17)	C22'—C21'—H21'	120.0
C11—C12—C15	121.06 (18)	C23'—C22'—C21'	120.0
C13—C12—C15	120.30 (18)	C23'—C22'—H22'	120.0
C12—C13—C8	121.29 (18)	C21'—C22'—H22'	120.0
C12—C13—H13	119.4	C22'—C23'—C24'	120.0
C8—C13—H13	119.4	C22'—C23'—H23'	120.0
C10—C14—H14A	109.5	C24'—C23'—H23'	120.0
C10—C14—H14B	109.5	C23'—C24'—C19'	120.0
H14A—C14—H14B	109.5	C23'—C24'—H24'	120.0
C10—C14—H14C	109.5	C19'—C24'—H24'	120.0
H14A—C14—H14C	109.5		
			
C6—N1—C1—O1	176.73 (18)	C15—C12—C13—C8	177.89 (18)
C6—N1—C1—C2	−2.7 (3)	C9—C8—C13—C12	0.6 (3)
O1—C1—C2—C5	−176.43 (18)	C7—C8—C13—C12	−176.36 (17)
N1—C1—C2—C5	3.0 (3)	C17—O3—C16—N2	75.77 (19)
O1—C1—C2—C3	3.4 (3)	C6—N2—C16—O3	−104.31 (19)
N1—C1—C2—C3	−177.20 (16)	C5—N2—C16—O3	72.9 (2)
C5—C2—C3—C4	97.0 (2)	C16—O3—C17—C18	144.73 (17)
C1—C2—C3—C4	−82.8 (2)	C19—O4—C18—C17	−170.9 (7)
C1—C2—C5—N2	−0.8 (3)	C19'—O4—C18—C17	−167.2 (6)
C3—C2—C5—N2	179.36 (17)	O3—C17—C18—O4	−64.1 (2)
C1—C2—C5—C7	179.49 (17)	C19'—O4—C19—C20	−35 (10)
C3—C2—C5—C7	−0.3 (3)	C18—O4—C19—C20	1.0 (12)
C6—N2—C5—C2	−2.0 (3)	C19'—O4—C19—C24	142 (11)
C16—N2—C5—C2	−179.04 (17)	C18—O4—C19—C24	177.9 (3)
C6—N2—C5—C7	177.74 (16)	O4—C19—C20—C21	176.7 (12)
C16—N2—C5—C7	0.7 (2)	C24—C19—C20—C21	0.0
C1—N1—C6—O2	−178.95 (17)	C19—C20—C21—C22	0.0
C1—N1—C6—N2	0.1 (3)	C20—C21—C22—C23	0.0
C5—N2—C6—O2	−178.63 (17)	C21—C22—C23—C24	0.0
C16—N2—C6—O2	−1.5 (3)	C22—C23—C24—C19	0.0
C5—N2—C6—N1	2.3 (3)	O4—C19—C24—C23	−177.1 (10)
C16—N2—C6—N1	179.49 (16)	C20—C19—C24—C23	0.0
C2—C5—C7—C8	−104.9 (2)	C19—O4—C19'—C20'	156 (11)
N2—C5—C7—C8	75.4 (2)	C18—O4—C19'—C20'	10.5 (10)
C5—C7—C8—C9	35.4 (2)	C19—O4—C19'—C24'	−25 (10)
C5—C7—C8—C13	−147.79 (18)	C18—O4—C19'—C24'	−170.5 (4)
C13—C8—C9—C10	−0.2 (3)	C24'—C19'—C20'—C21'	0.0
C7—C8—C9—C10	176.65 (18)	O4—C19'—C20'—C21'	178.9 (11)
C8—C9—C10—C11	0.4 (3)	C19'—C20'—C21'—C22'	0.0
C8—C9—C10—C14	−179.14 (17)	C20'—C21'—C22'—C23'	0.0
C9—C10—C11—C12	−1.0 (3)	C21'—C22'—C23'—C24'	0.0
C14—C10—C11—C12	178.52 (18)	C22'—C23'—C24'—C19'	0.0
C10—C11—C12—C13	1.4 (3)	C20'—C19'—C24'—C23'	0.0
C10—C11—C12—C15	−177.69 (19)	O4—C19'—C24'—C23'	−179.0 (10)
C11—C12—C13—C8	−1.2 (3)		

### Hydrogen-bond geometry (Å, °)

**Table:**

*D*—H···*A*	*D*—H	H···*A*	*D*···*A*	*D*—H···*A*
N1—H1···O2^i^	0.92 (3)	1.94 (3)	2.859 (2)	173 (2)

Symmetry codes: (i) −*x*+1, −*y*+2, −*z*+1.
